# Etiology and Prevention of an Endodontic Iatrogenic Event: Instrument Fracture

**DOI:** 10.25122/jml-2020-0137

**Published:** 2020

**Authors:** Oana Amza, Bogdan Dimitriu, Ioana Suciu, Ruxandra Bartok, Mihaela Chirila

**Affiliations:** 1. Department of Endodontics, “Carol Davila” University of Medicine and Pharmacy, Bucharest, Romania; 2. Department of Aesthetics in Dental Medicine, “Carol Davila” University of Medicine and Pharmacy, Bucharest, Romania

**Keywords:** Endodontic instrumentation, instrument separation, etiology, prevention

## Abstract

Although benefiting from an unprecedented technological evolution, contemporary endodontics is still characterized by the recurrence of retreatments, due to the need to solve quite frequent incidents, accidents, or even failures of primary endodontic treatment. This survey aims to assess both the etiology and the prevention methods of one of the most troublesome endodontic iatrogenies: instrument separation during root canal shaping. The multifactorial nature of this occurrence entails identifying and taking into account all the causal and contributing factors. Their significant number and the possible involvement of any of them, starting with the complexity and variability of the root canals and ending with the technical specifications concerning the nickel-titanium rotary instrumentation system being used, highlight the necessity to develop valid guidelines to avert the occurring of such an upsetting situation.

## Introduction

Present-day endodontics witnesses a plethora of developments as the result of continuous scientific evolution and advancements in technologies, methods, and materials. These materialize in every aspect of root canal treatment, from diagnostic imaging investigations, designing and kinematics of instrumentation, to different materials and techniques concerning root canal filling. The results make it possible to approach treatment options for complex clinical situations, with the outcome of healed teeth, i.e., “functional, asymptomatic teeth with no or minimal radiographic periradicular pathosis” [1].

However, endodontic procedures may frequently be challenging, with an important number of issues having consequences on the degree of difficulty and the risks involved by the treatment option required. In turn, these must be correctly evaluated, beginning with an accurate diagnosis and a reasonable prognosis to achieve a comprehensive treatment plan. Aspects that are unique to each case have to be considered, such as the complexity of the root canal anatomy. Evidence provided by thorough clinical examination and diagnostic imaging enables a correct appraisal of preoperative diagnosis and treatment planning in endodontics [2].

However, a significant number of incidents or accidents may occur during endodontic treatment. Since 2002, these are considered to be “unfortunate occurrences that happen during treatment, some owing to inattention to detail, others totally unpredictable” [3]. Such accidents may arise in any endodontic treatment stage and have the potential to generate treatment failure. Later on, Walton and Torabinejad defined endodontic mishaps as “unwanted or unforeseen circumstances during root canal therapy that can affect the prognosis” [4].

Endodontic mishaps are numerous and diverse and are usually classified according to:

• John Ingle: access-related, instrumentation-related, obturation-related and miscellaneous mishaps;
• Walton & Torabinejad: procedural accidents during access preparation, accidents during cleaning and shaping, accidents during obturation and accidents during post space preparation;
• Leif Tronstad: incomplete analgesia, access cavity, perforations from the pulp chamber, root perforations, obliterated root canal, fracture of an instrument, adverse reactions to medicaments, overfilling of the root canal and vertical root fractures [5].

It is hard to evaluate the real frequency of occurrence of these mishaps rigorously. A significant number of clinical cases is suspected to be overlooked, either by not observing or not reporting them.

All of the above-mentioned possible mishaps include fracture of an instrument, also known as instrument separation.

### Etiology of endodontic instrument separation

The fracture of an instrument is a very disturbing situation, a barrier being thus raised in front of further shaping and filling that particular root canal. Apical access to that obstruction is denied, and root canal treatment cannot be completely performed.

Depending on both the moment during the endodontic treatment when the accident happened, and the level of the root canal where the instrument broke, proper shaping and filling of the root canal is more or less prevented. This leads to possible complications and may even endanger the positive outcome of the entire endodontic treatment. Starting with Strindberg in 1956, an important number of surveys approached the matter from both clinical and statistical viewpoints [6].

Research has shown that the incidence of endodontic files separation is extremely variable, strongly depending on some factors influencing how statistics surveys was performed [7-10], such as the type of study (in vitro or in vivo), the number of teeth or to the number of root canals, the clinician’s experience (endodontist, endodontics resident, general practitioner), the kind of teeth taken into account, studies generally focusing on molars, the kind of endodontic instruments (both hand and rotary files for older studies, exclusively rotary files for studies over the last two decades), the type of rotary system, the type of imaging investigations (X-ray, CBCT or both).

It is important to note that any endodontic instrument may fracture during its use: endodontic probes, Gates Glidden drills, stainless steel files, nickel-titanium files, irrigation tips, ultrasonic tips, spreaders, pluggers, and others. The most common situations involve endodontic files separated during root canal shaping. 

It is interesting to mention that the fracture incidence of rotary nickel-titanium (Ni-Ti) instruments was significantly greater during root canal re-treatment than root canal treatment, mainly with small instruments and in the apical third of the canal [11].

From a statistical perspective, the incidence concerning the fracture of an endodontic instrument is mainly influenced by the way in which these studies are carried out; thus, a wide range of values could be encountered:

• Stainless steel files: 0.7 - 7.4%;
• NiTi rotary files driven by continuous rotation: 0.4 - 5%;
• NiTi rotary files driven by reciprocation: 0.14%;
• SAF (Self Adjusting File): 0.6%.

This reveals that the frequency of nickel-titanium rotary instruments fracture is comparable to that of stainless-steel endodontic files, although the metallurgical and mechanical properties of nickel-titanium alloy are definitely superior, mainly due to its superelasticity. Even more, there are studies suggesting a higher incidence of fracture for NiTi rotary instruments compared with stainless steel files.

It should also be noted that, unlike NiTi instruments, the stainless-steel ones could present some “warnings” of imminent breakage, represented by plastic deformations of the file: unwidening, overwidening or angulations at sharp angles.

However, the fracture mechanisms are different for the two different kinds of endodontic instruments: excessive amounts of torque for stainless-steel files and mainly torsion overload and cyclic fatigue for the nickel-titanium ones [12].

The fracture of the endodontic instrument has a multifactorial etiology, being influenced by diverse elements [13, 14] such as the characteristics of the access cavity, the geometry of root canals, the cross-sectional features of the root canals, which, in turn, is influenced by the endodontic pathology and age of the patient, the design features of rotary instruments, metallurgical properties of various nickel-titanium rotary instruments, imperfections or manufacturing defects of the instrument, the instrumentation technique, instrument dynamics in the root canal, the number of sterilization cycles to which the instrumentation has been subjected and its number of uses, the difficulty of the previous clinical situations that the instrument has been already subjected to, previous endodontic treatments and the clinician’s experience.

### Prevention of endodontic instrument separation

The endodontic access cavity must allow a direct view of all root canal orifices and straight-line access to the first curvature of each root canal, considering the root canal is accessible over its entire working length.

The absence of complete removal of the ceiling of the pulp chamber, as well as a restriction of straight-line access, constitute the main reasons for higher stress of the rotary file resulting in an increased risk of fracture.

Endodontic instrumentation, currently exclusively based on nickel-titanium rotary systems, benefits from the illumination and magnification provided by the endodontic operating microscope, which also allows the implementation of the concept of minimally invasive access cavities. Studies conducted in this respect do not indicate an increase in the incidence of endodontic instrument fracture, which shows that root canal shaping can also be performed with minimal risks under the specific conditions of minimally invasive dentistry under the conditions offered by endodontic specialists and current treatment options [14-16].

The importance of root canal anatomy is evident because the risk of instrument fracture increases directly proportional to the complexity of the endodontic system. Most cases occur in both upper and lower molars, and more precisely, in their mesiobuccal root canals [17]. The explanation lies, in particular, in the existing curvatures of these root canals [18, 19].

Each curvature is described by two parameters: its angle and its radius, and the two elements are independent of each other. At the same angulation, two root canals may have different radii of curvature. The smaller the radius, the sharper the curvature. The rotating file is subjected to repeated stress cycles - exerted on its external curvature - and compression cycles - on its internal curvature. With the increase of the angulation and the reduction of the radius, the instrument is increasingly stressed by the increasing torsional values. From this perspective, the dimensions of the radius of curvature seem to be more important.

The succession of these cycles, in the conditions of tight curvatures, or even several successive curvatures along the root canal, causes an increase of the fracture risk of the instrument. 

Consequently, the main recommendation is to use a continuous axial movement of the instrument during its rotation, thus avoiding the concentration of the stresses only in certain places of the file. 

The cross-section of root canals also plays an important role [17]. On the one hand, it may be very far from the ideal circular, being flattened, polylobulated, or irregular. On the other hand, the shape of the cross-section may vary along the root canal. There may also be narrowing areas and, of course, partial or totally inaccessible root canals as a consequence of endodontic pathology and aging. All these situations create the premises of blocking, torsion, and fracture of the file. An instrument can be fractured at any level of the root canal with a higher prevalence at the curvatures and the apical third [18].

In any type of rotation, there are two parameters in inverse proportionality: rotational speed and torque [18, 19]. The latter diminishes as the speed increases, thus compensating for the negative effect of speed increase on the development of cyclical fatigue. Therefore, the current trend is to use reduced speeds to minimize the risk of fracture.

Effective and safe instrumentation is mainly dependent on correct endodontic irrigation, in order to allow the removal of debris and to continuously lubricate the root canal.

Another essential parameter concerning the etiology of an endodontic instrument separation is the number of sterilization cycles to which it has been subjected, which is often indicated by the manufacturer.

The cumulative stress an endodontic file undergoes is dependent both quantitatively (the number of uses) and qualitatively (the difficulty of the prepared root canals). All the studies conducted in this regard demonstrate the direct proportionality between the number of uses of endodontic instruments and the incidence of their fracture.

There are also situations in which, considering that the mechanical stresses to which the instruments were subjected were very important, these should be discarded after their first use, otherwise significantly increasing the risk of fracture when reused.

## Conclusions

In relation to the above mentioned, the preventive elements regarding the endodontic instruments fracture can be summed up as follows:

• achieving an endodontic approach as close as possible to the right line, avoiding the excessive curvature of the instrument;
• verifying the access of the root canals throughout the entire working length;
• obtaining a sliding path that allows torque to be maximally effective;
• endodontic irrigation present throughout the instrumentation, with the help of effective systems, which allow removal of the debris throughout the length of the root canal (Endovac, for example);
• the use of current rotary systems with minimal engagement and minimal “screw-in” effect in dentin;
• observing the speed and torque values indicated by the manufacturer for each specific type of rotary system;
• minimizing the number of uses of endodontic files or implementing rotary systems that allow a single use of an instrument - “One Endo file”.

Though any endodontic instrument may break in any given case, an accurate assessment of the clinical and imaging diagnosis, comprehensive knowledge concerning the instruments being used, as well as consistently relying on the operating microscope can provide significant means in order to avoid a troublesome experience and perform the best endodontic treatment possible.

## Conflict of Interest

The authors declare that there is no conflict of interest.

## References

[1] AAE. (2020). Glossary of Endodontic Terms.

[2] AAE (2018). The Impact of Cone Beam Computed Tomography in Endodontics: A New Era in Diagnosis and Treatment Planning.

[3] Frank RJ. (2002). Chapter 14 Endodontic mishaps: their detection, correction, and prevention. in Endodontics.

[4] Torabinejad M., Walton RE, Fouad AF (2015). Endodontics: principles and practice.

[5] Tronstad L. (2008). Clinical Endodontics. A Textbook.

[6] Strindberg LZ. (1956). The dependence of the results of pulp therapy on certain factors: an analysis study based on radiographic and clinical follow up examination. Acta Odontol Scand.

[7] Cunha RS., Junaid A, Ensinas P, Nudera W, Bueno CE (2014). Assessment of the separation incidence of reciprocating WaveOne files: a prospective clinical study. J Endod.

[8] Bueno CSP., Oliveira DP, Pelegrine RA (2020). Fracture incidence of WaveOne Gold files: a prospective clinical study [published online ahead of print, 2020 Jun 23]. Int Endod J.

[9] Caballero-Flores H., Nabeshima CK, Binotto E, Machado MEL (2019). Fracture incidence of instruments from a single-file reciprocating system by students in an endodontic graduate programme: a cross-sectional retrospective study. Int Endod J.

[10] Coelho MS., Rios MA, Bueno CEDS (2018). Separation of Nickel-Titanium Rotary and Reciprocating Instruments: A Mini-Review of Clinical Studies. Open Dent J.

[11] Alfouzan K., Jamleh A (2018). Fracture of nickel titanium rotary instrument during root canal treatment and re-treatment: a 5-year retrospective study. Int Endod J.

[12] Bahcall JK. (2013). Remedying and preventing endodontic rotary nickel-titanium (NiTi) file breakage. Compend Contin Educ Dent.

[13] Khasnis SA., Kar PP, Kamal A, Patil JD (2018). Rotary science and its impact on instrument separation: A focused review. J Conserv Dent.

[14] Lambrianidis T. (2018). Management of Fractured Endodontic Instruments. A Clinical Guide.

[15] Mullane EM. (2015). Tips to avoid instrument separation in endodontics. J Ir Dent Assoc.

[16] Moore B., Verdelis K, Kishen A, Dao T, Friedman S (2016). Impacts of contracted endodontic cavities on instrumentation efficacy and biomechanical responses in maxillary molars. J Endod.

[17] Vouzara T., el Chares M, Lyroudia K (2018). Separated Instrument in Endodontics: Frequency, Treatment and Prognosis. Balk J Dent Med.

[18] Eaton JA., Clement DJ, Lloyd A, Marchesan MA (2015). Micro-computed tomographic evaluation of the influence of root canal system landmarks on access outline forms and canal curvatures in mandibular molars. J Endod.

[19] Roda SR., Gettleman BH, Hargreaves K, Berman LH (2016). Nonsurgical retreatment. Cohen’s pathways of the pulp.

